# Examining the effectiveness of artificial intelligence applications in asthma and COPD outpatient support in terms of patient health and public cost: SWOT analysis

**DOI:** 10.1097/MD.0000000000038998

**Published:** 2024-07-19

**Authors:** Seha Akduman, Kadir Yilmaz

**Affiliations:** a Department of Pulmonary Diseases, Yeditepe University, Faculty of Medicine, Istanbul, Türkiye; b Istanbul Commerce University, Social Sciences Institute, Industrial Policies and Technology Management Program (DR), Istanbul, Türkiye.

**Keywords:** artificial intelligence, asthma, COPD, outpatient treatment

## Abstract

This research aimed to examine the effectiveness of artificial intelligence applications in asthma and chronic obstructive pulmonary disease (COPD) outpatient treatment support in terms of patient health and public costs. The data obtained in the research using semiotic analysis, content analysis and trend analysis methods were analyzed with strengths, weakness, opportunities, threats (SWOT) analysis. In this context, 18 studies related to asthma, COPD and artificial intelligence were evaluated. The strengths of artificial intelligence applications in asthma and COPD outpatient treatment stand out as early diagnosis, access to more patients and reduced costs. The points that stand out among the weaknesses are the acceptance and use of technology and vulnerabilities related to artificial intelligence. Opportunities arise in developing differential diagnoses of asthma and COPD and in examining prognoses for the diseases more effectively. Malicious use, commercial data leaks and data security issues stand out among the threats. Although artificial intelligence applications provide great convenience in the outpatient treatment process for asthma and COPD diseases, precautions must be taken on a global scale and with the participation of international organizations against weaknesses and threats. In addition, there is an urgent need for accreditation for the practices to be carried out in this regard.

## 1. Introduction

Asthma and chronic obstructive pulmonary disease (COPD) are among the most common respiratory diseases and are 2 very important diseases in terms of individuals’ quality of life and public costs.^[1,2]^ Since asthma and COPD are similar in terms of symptoms, there may be some confusion and incorrect treatment in the diagnosis and treatment processes of these illness.^[3–5]^ Although the number of studies conducted in this field increases, it is possible to state that a large part of society is affected by COPD and asthma diseases.^[6–8]^ Increased risk factors for disease, dietary and lifestyle choices, physical activity levels, and lifestyle problems have all contributed to the rise in these illnesses in recent years.

Although outpatient treatment is possible in asthma and COPD diseases in the early stages of the disease and early diagnosis, inpatient treatment is required in advanced stages and worsening clinical pictures of the disease.^[9–11]^ In this regard, outpatient treatment is important both in the early diagnosis phase and in reducing the number of cases requiring hospitalization.^[12,13]^ Outpatient treatment is also important in monitoring the prognosis and causative factors of the disease in asthma and COPD patients.^[14,15]^ However, regarding these 2 diseases and the course of the disease, there may be some disruptions in the outpatient treatment process.

The use of digital technologies in medical services and health sciences has provided significant gains in the fight against diseases and public health.^[16,17]^ Information technologies have made significant contributions to many processes, from recording and compiling diseases and causative processes to analysis and advanced inferences.^[18,19]^ Among these, artificial intelligence applications, which have come to the fore in recent years and are generally based on machine learning, come to the fore. Artificial intelligence, in its simplest and broadest definition, is the ability of codes and software produced in the computer environment to receive statistical information from big data, understand them, classify them, compile them and make inferences from them according to the desired outputs.^[17–19]^ The ability to learn and the power to make inferences, which are the most important features of artificial intelligence, enable artificial intelligence-based applications to find more and more areas of use today.

Though artificial intelligence is being used in the medical field in numerous studies and real-world applications, not enough research has been done on how, where, and how much artificial intelligence will be used in outpatient treatments, as well as what benefits it will bring to the field and the patient treatment process. Therefore, this study aimed to examine the effectiveness of artificial intelligence applications in asthma and COPD outpatient treatment support in terms of patient health and public cost.

## 2. Methods

### 2.1. Research model

The research was designed in descriptive scanning and an embedded pattern model. In this design, the researcher can evaluate any subject or phenomenon by using content analysis and semiotic analysis methods on secondary data.^[20,21]^ In the descriptive scanning model, the researcher compiles information about the current situation on the relevant subject and enables the situation to be described from various perspectives.^[22]^ In the study, resources and studies on artificial intelligence applications—a relatively new phenomenon—as well as the field of outpatient treatment for COPD and asthma were looked at, compiled, and then presented with an embedded pattern. Based on the results and literature reviews, a description of the research was then provided.

### 2.2. Data collection and analysis

Content analysis, document scanning analysis and semiotic analysis methods were used to collect research data. In this context, indicators of strengths, weaknesses, opportunities and threats were determined and their frequency analysis was carried out. 18 studies related to asthma, COPD and artificial intelligence were evaluated from Web of Science and Scopus databases.^[23–40]^ In the semiotic analysis, the distribution of indicators for each field is shown in Table 1.

**Table 1:** Indicators used in research analysis.

Strengths	Weaknesses	Opportunities	Threats
Easy access	Misdiagnosis	Easy access	Unreliable information
Effective evaluation	Systematic error	Public cost	Malicious use
Big data and sampling	Wrong relationship	Simultaneous evaluation	Low diagnostic sensitivity
Differential diagnosis	Differential criteria	Treatment development	Individual misconceptions
Awareness raising	Open source and security	Do not understand the reason	Manipulations

For the findings obtained from semiotic analyzes in the analysis of the data, indicators were selected with the help of the Maxquda 2022 package program and then analyzed by the frequency analysis method in the Microsoft Excel package program.

**Figure 1.**
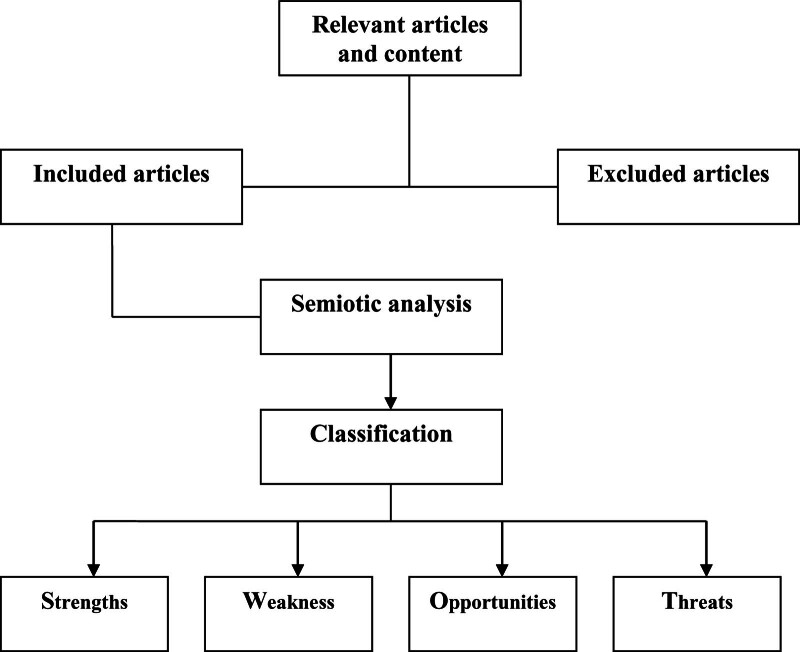
Research protocol flowchart.

Strengths, weakness, opportunities, threats (SWOT) analysis was used in the qualitative analysis phase of the research. SWOT analysis is a method in which strengths, weaknesses, opportunities and threats about any phenomenon are analyzed. The method takes its name from the first letters of these words. In SWOT analysis, the researcher makes use of sources such as interviews, surveys and literature studies on the subject when making judgments about a phenomenon or content.^[20–22]^ In the research, SWOT analysis was analyzed using qualitative methods and semiotic analysis methods of literature studies that went through the referee process and received approval. After semiotic analysis, scientific articles and findings were classified and then evaluated according to SWOT analysis (Fig. 1).

Validity of SWOT analysis is discussed and accepted in literature as a semi-quantitative approach.^[20–22]^ Quantitative and qualitative data are needed to make a judgment about a phenomenon or issue. Although quantitative data includes a reliability and validity method within itself, this is a bit difficult in qualitative or semi-qualitative methods. On the other hand, in SWOT analysis, a high level of validity was accepted when the arguments used were supported by literature studies.^[20–22]^ In addition, all qualitative information required for SWOT analysis was obtained from accepted scientific studies in Web of Science and peer-reviewed journals.

## 3. Results

In the research, both the findings obtained from semiotic analysis and content analysis and the findings obtained as a result of trend analysis were evaluated together, and the results obtained were given under separate headings in this section.

### 3.1. Strengths

One of the main strengths of using artificial intelligence in asthma and COPD outpatient treatment is working with big data and therefore having high statistical sample power. In addition, there are strengths such as shorter access to outpatient treatment methods and the evaluation of more cases at the same time, which can be listed as follows^[23–40]^:

− Access to asthma and COPD patients in a shorter time in cases requiring outpatient treatment,
− Evaluation of other disease factors that may be associated with asthma and COPD,
− More effective evaluation of symptom differences and patient histories between asthma and COPD,
− Allows evaluation through big data during the diagnosis process, instead of the number of patients in a single doctor professional experience,
− In fact, distinguishing between people who have asthma or COPD and those who do not come to the hospital and thus increasing the individual quality of life,
− Reduction of misdiagnoses or incomplete diagnoses regarding asthma and COPD,
− Allowing individuals to avoid self-stigma and misinformation regarding asthma and COPD.

The frequency analysis results of the publications examined for strengths are given in Table 2.

**Table 2:** Frequency analysis results for strengths.

Strengths	Number of publications (n)	Percentage (%)
Easy access	12	66.7
Effective evaluation	10	55.6
Big data and sampling	8	44.4
Differential diagnosis	6	33.3
Awareness raising	4	22.2

Easy access was found in 66.7% of the studies examined, effective evaluation in 55.6%, big data and sample in 44.4%, differential diagnosis in 33.3% and awareness-raising indicators in 22.2%.

### 3.2. Weakness

The main weaknesses of asthma and COPD outpatient treatment are the deficiencies of artificial intelligence applications and the resulting software problems and especially the weaknesses arising from the limiting points of artificial intelligence. It is possible to list them as follows^[23–40]^:

− Due to the similarity of asthma and COPD symptoms, the possibility of misdiagnosis also exists in artificial intelligence,
− Negatively affecting the diagnostic process due to possible systematic errors regarding the asthma and COPD treatment process,
− Data on the relationship between asthma and COPD and comorbidities are open to misinterpretation,
− Despite the differences in the treatment process of asthma and COPD, the diagnostic process and the distinguishing criteria between symptoms are prone to confusion.
− Since the use of the system is open to everyone and opens data access, it is vulnerable to serious contamination in big data,
− Open source data is open to manipulation and disinformation,
− It appears as an alternative intended to replace medical units and treatment processes and is susceptible to malicious uses.

The frequency analysis results of the publications examined for weaknesses are given in Table 3.

**Table 3:** Frequency analysis results for weaknesses.

Weaknesses	Number of publications (n)	Percentage (%)
Misdiagnosis	14	77.8
Systematic error	8	44.4
Wrong relationship	6	33.3
Differential criteria	4	22.2
Open source and security	3	16.7

Misdiagnosis was found in 77.8% of the studies examined, systematic errors in 44.4%, incorrect association in 33.3%, differential criteria in 22.2%, and open source and security indicators in 16.7%.

### 3.3. Opportunities

It is possible to consider the opportunities for asthma and COPD outpatient treatment together with the opportunities that artificial intelligence applications can bring to the field of healthcare in general. Among these, there are many opportunities, from reducing the costs of health services to providing health services to more people, to increasing diagnostic accuracy with evaluations based on big data about diseases. It is possible to list them as follows^[23–40]^:

− Easier access to health services and accessibility to wider segments,
− Reducing public costs and burden in health services,
− More cases can be evaluated together and simultaneously,
− Determining treatment processes by taking into account the interaction and simultaneous effects of multiple risk factors in cases,
− Ability to analyze the relationship between previous treatment processes and the current situation in a multidimensional way,
− Identifying external factors that cause diseases in different societies and social structures,
− Evaluating the differences in disease-causing factors according to geographical and social regions, with the help of cross-comparisons of data.

The frequency analysis results of the publications examined for opportunities are given in Table 4.

**Table 4:** Frequency analysis results for opportunities.

Opportunities	Number of publications (n)	Percentage (%)
Easy access	11	61.1
Public cost	10	55.6
Simultaneous evaluation	8	44.4
Treatment development	8	44.4
Understanding the cause	4	22.2

Indicators of easy access were found in 61.1% of the studies examined, public cost in 55.6%, simultaneous evaluation in 44.4%, treatment development in 44.4%, and understanding the cause in 22.2%.

### 3.4. Threats

Similar to opportunities, the most important threats to artificial intelligence applications in asthma and COPD outpatient care come from the threats of artificial intelligence in general. In addition to threats, it is possible to state that the following threats are also valid for asthma and COPD outpatient treatment, as the system works on big data, machine learning and data limitations, and the limiting factors have low levels of determination^[23–40]^:

− Leakage of unverified and unreviewed information on the internet into the artificial intelligence system,
− Misdiagnosis and inappropriate and unnecessary treatments are given due to not determining sufficient limiting factors related to COPD and asthma,
− Providing manipulative data to data providers for malicious use of the system,
− Due to its vulnerability to systematic errors, sensitivity and specificity levels decrease and false positive and negative rates increase,
− Using the system for different purposes on a national and international scale,
− Developing individuals’ perceptions that there is no need for basic health services,
− Healthcare costs become untrackable and maliciously manipulative.

The frequency analysis results of the publications examined for threats are given in Table 5.

**Table 5:** Frequency analysis results for threats.

Threats	Number of publications (n)	Percentage (%)
Unreliable information	15	83.3
Malicious use	11	61.1
Low diagnostic sensitivity	9	50.0
Individual misconceptions	7	38.9
Manipulations	5	27.8

Indicators of unreliable information were found in 83.3% of the studies reviewed, malicious use in 61.1%, low diagnostic sensitivity in 50.0%, individual misconceptions in 38.9%, and manipulations in 27.8%.

## 4. Discussion

In this research, it was aimed to examine the strengths, weaknesses, opportunities and threats of the use of artificial intelligence applications in the asthma and COPD outpatient treatment process, and within this framework, the current situation of artificial intelligence in the asthma and COPD outpatient treatment process was analyzed by making semiotic analysis through literature studies.

There are limited studies in the literature on the use and benefits of digital technologies for asthma and COPD.^[23–40]^ These studies generally report that digital technologies make significant contributions to the diagnosis and treatment process of asthma and COPD.^[16–19]^ Based on the findings of our study, the most valued strength is accessibility, which is followed by efficient assessment, large data and sample, differential diagnosis, and growing awareness. Artificial intelligence applications in asthma and COPD are generally strong because they can reach a larger patient base and improve the quality of healthcare for a greater number of people. In addition, evaluations based on big data in artificial intelligence applications also show that artificial intelligence data-based studies have, in a sense, converged to the quality of Phase 3 studies. However, the weaknesses and threats of big data need to be well analyzed and understood.

Outpatient studies on asthma and COPD indicate that the symptoms of both diseases are similar and therefore the differential diagnosis is weak. Although there is no study on direct artificial intelligence applications in asthma and COPD outpatient treatment, it is reported that artificial intelligence applications in the outpatient phase have some weaknesses, especially health literacy. The most prominent indicator of weaknesses is misdiagnosis, followed by systematic error, incorrect association, differential criteria and security vulnerabilities arising from open source. The weaknesses of using artificial intelligence in the outpatient treatment of asthma and COPD diseases are, in general, that the system still has many deficiencies and unknowns, and therefore, for both diseases, there will be some deficiencies in the diagnosis and treatment phase, physical examination, physician evaluation and evaluation with history. However, it is possible to reduce these weaknesses by providing public support through comprehensive field and scientific studies.

The opportunities of outpatient treatment of asthma and COPD diseases reveal the public duty and the ability to reach more segments, which is the most basic feature of health in general. The indicator with the highest frequency regarding opportunities is easy access, followed by public cost, simultaneous evaluation, treatment development and cause. In addition, by creating national, regional and global databases, it is possible to ensure an all-out, global fight against asthma and COPD diseases. However, in order for these opportunities to turn into strengths, weaknesses and threats must first be emphasized, and the use of the system must be evaluated in terms of possible vulnerabilities and undesirable consequences.

Although outpatient studies on asthma and COPD do not specifically mention threats to these 2 diseases, threats are generally mentioned in the diagnostic processes of artificial intelligence applications. The most prominent among these are data security and awareness. According to our research results, unreliable information is the leading indicator of threats, followed by malicious use, low diagnostic sensitivity, individual misconceptions and manipulations.

Although the results obtained in the research seem useful in the outpatient treatment process of artificial intelligence for asthma and COPD, some critical parameters should also be mentioned. These are the issues that arise from the threats and weaknesses in the SWOT analysis, especially the use of technology for profit or malicious purposes, data security and criminality. Especially data security and data manipulation are the most basic and critical parameters that should be emphasized at this point.

More innovative solutions can be introduced to prevent critical malicious uses in this regard. There is a need for new innovative systems that will enable doctors to share data and information, especially with a full understanding of the components of the outpatient treatment process and data security as a whole.

Although it is a pioneer in the field, it is possible to state that new studies have been put forward on the use of artificial intelligence in the field of medicine. Among these Hastimoto et al evaluated role of artificial intelligence on anesthesiology, and reported similar results as there are benefits and cautions.^[41]^ In another study, Hunter et al evaluated role of artificial intelligence on cancer diagnosis.^[42]^ Ji et al reported that artificial intelligence may help diagnosis of retinal vascular diseases.^[43]^ The common point of all these studies is that although there are some obstacles and threats, artificial intelligence is important and beneficial in the medical sense.

### 4.1. Limitations of the study

The main limitations of the research are the uncertainties regarding artificial intelligence and the lack of predictions regarding the applications and studies to be carried out in this field. Although digital technologies have a relatively long history in healthcare, artificial intelligence is a fairly new concept. In addition, the development rate of studies on artificial intelligence has a very high acceleration compared to digital technologies. For this reason, like all studies conducted in the field of artificial intelligence, the most important limitation in this research is incomplete information about artificial intelligence and issues that need research.

Another important limitation of the study is that there is not sufficient and comprehensive data regarding the outpatient treatment process regarding asthma and COPD. Although asthma and COPD are 2 important diseases that are very common in society, there are many variables regarding their diagnosis processes, follow-up and prognosis processes of diagnosed cases. For this reason, the research was conducted on qualitative data rather than quantitative data. This is another important limitation of the research.

### 4.2. Contributions of the research to the literature and the field

The primary contribution of the research to the field is that it is a pioneer in the field by bringing a different perspective of use of artificial intelligence applications in the field of health. Although the literature focuses on the pragmatic and field aspects of artificial intelligence and its applications in the field of health, there are not enough studies that look at the issue in a general and broad context, in a way that will also contribute to the public. Therefore, it is possible to argue that the research is pioneering in the field and contributes to the literature.

The important contribution of the research to the field is that it includes some results as a warning for future studies in terms of public health, by addressing the possible risks and weaknesses of artificial intelligence applications. Today, studies on artificial intelligence are more focused on emerging in practice in the fastest and most profitable way. However, the research also reveals weaknesses and threats as a warning to the studies done and to be done in the field.

## 5. Conclusion

Asthma and COPD outpatient treatment brings many benefits, from helping individuals have a healthy life and improving their quality of life to reducing public health costs. In this process, artificial intelligence applications in asthma and COPD outpatient treatment undoubtedly present significant strengths and many opportunities. However, in addition to these strengths and opportunities, weaknesses and threats are also very important. As revealed in the mostly qualitative and pioneering studies conducted in this field, artificial intelligence applications have the potential to lead to very serious and undesirable consequences with higher public costs, from manipulation to disinformation, from misinformation to the relegation of medical processes to the background.

In order for artificial intelligence applications to be used more effectively in asthma and COPD outpatient treatment, there is a need to first eliminate the security and open source features of these technologies, evaluate the limitations of the systems, and new guideline studies with the participation of large international organizations and parties. It is also beneficial to conduct further research on the use of big data for artificial intelligence applications in terms of differential treatment processes and prognosis, especially for asthma and COPD.

## Author contributions

**Conceptualization:** Seha Akduman.

**Data curation:** Seha Akduman.

**Formal analysis:** Seha Akduman, Kadir Yilmaz.

**Methodology:** Kadir Yilmaz.

**Writing – original draft:** Seha Akduman, Kadir Yilmaz.

**Writing – review & editing:** Seha Akduman, Kadir Yilmaz.

## References

[1] Barnes PJ. Mechanisms in COPD: differences from asthma. Chest. 2000;117:10S–4S. 10673467 10.1378/chest.117.2_suppl.10s

[2] Gibson PG McDonald VM. “Asthma–COPD overlap 2015: now we are six.”. Thorax. 2015;70:683–91. 25948695 10.1136/thoraxjnl-2014-206740

[3] Postma DS Rabe KF. “The asthma–COPD overlap syndrome.”. N Engl J Med. 2015;373:1241–9. 26398072 10.1056/NEJMra1411863

[4] Cosio BG Soriano JB López-Campos JL. “Defining the asthma-COPD overlap syndrome in a COPD cohort.”. Chest. 2016;149:45–52. 26291753 10.1378/chest.15-1055

[5] Barnes PJ. “Asthma-COPD overlap.”. Chest. 2016;149:7–8. 26757281 10.1016/j.chest.2015.08.017

[6] Mannino DM. COPD: epidemiology, prevalence, morbidity and mortality, and disease heterogeneity. Chest. 2002;121:121S–6S. 12010839 10.1378/chest.121.5_suppl.121s

[7] Mendy A Forno E Niyonsenga T. “Prevalence and features of asthma-COPD overlap in the United States 2007–2012.”. Clin Respirat J. 2018;12:2369–77. 10.1111/crj.12917 PMC6287748 29873189

[8] De Marco R Pesce G Marcon A. “The coexistence of asthma and chronic obstructive pulmonary disease (COPD): prevalence and risk factors in young, middle-aged and elderly people from the general population.”. PLoS One. 2013;8:e62985. 23675448 10.1371/journal.pone.0062985 PMC3651288

[9] Bellocq A Gaspard W Couffignal C. “Outpatient pulmonary rehabilitation for severe asthma with fixed airway obstruction: comparison with COPD.”. J Asthma. 2019;56:1325–33. 30693816 10.1080/02770903.2018.1541351

[10] Rootmensen GN van Keimpema ARJ Looysen EE. “The effects of additional care by a pulmonary nurse for asthma and COPD patients at a respiratory outpatient clinic: results from a double blind, randomized clinical trial.”. Patient Educ Couns. 2008;70:179–86. 18031971 10.1016/j.pec.2007.09.021

[11] Sutherland ER. “Outpatient treatment of chronic obstructive pulmonary disease: comparisons with asthma.”. J Allergy Clin Immunol. 2004;114:715–24; quiz 725. 15480305 10.1016/j.jaci.2004.07.044

[12] Riley CM Sciurba FC. “Diagnosis and outpatient management of chronic obstructive pulmonary disease: a review.”. JAMA. 2019;321:786–97. 30806700 10.1001/jama.2019.0131

[13] Al-Zahrani JM Ahmad A Al-Harbi A. “Factors associated with poor asthma control in the outpatient clinic setting.”. Ann Thor Med. 2015;10:100–4. 10.4103/1817-1737.152450 PMC4375737 25829960

[14] Ali Z Dirks CG Ulrik CS. “Long-term mortality among adults with asthma: a 25-year follow-up of 1,075 outpatients with asthma.”. Chest. 2013;143:1649–55. 23471206 10.1378/chest.12-2289

[15] Gutiérrez FJA Galván MF Gallardo JFM. “Predictive factors for moderate or severe exacerbations in asthma patients receiving outpatient care.”. BMC Pulm Med. 2017;17:1–7. 28464895 10.1186/s12890-017-0422-6 PMC5414178

[16] Senbekov M Saliev T Bukeyeva Z. “The recent progress and applications of digital technologies in healthcare: a review.”. Int J Telemed Appl. 2020;2020:8830200. 33343657 10.1155/2020/8830200 PMC7732404

[17] Mamyrbekova S Nurgaliyeva Z Saktapov A. “Medicine of the future: digital technologies in healthcare. E3S Web Conferences. 159. EDP Sciences, 2020. 04036.

[18] Bazarbayev M. Digital medical ecosystem: transformation and development prospects. Sci Innovation. 2023;2:64–9.

[19] Ter-Akopov GN Kosinova NN Knyazev SA. “Digital technologies in healthcare: achievements and prospects. 1st International Scientific Conference” Modern Management Trends and the Digital Economy: from Regional Development to Global Economic Growth”(MTDE 2019). Atlantis Press, 2019.

[20] Armitage P Berry G, and Matthews JNS. Statistical methods in medical research. New York: John Wiley & Sons, 2008.

[21] Hanson JL Balmer DF Giardino AP. “Qualitative research methods for medical educators.”. Acad Pediatr. 2011;11:375–86. 21783450 10.1016/j.acap.2011.05.001

[22] Yilmaz K Turanli M. A multi-disciplinary investigation of linearization deviations in different regression models. Asian J Probab Stat. 2023;22:15–9.

[23] Kaplan A Cao H FitzGerald JM. Artificial intelligence/machine learning in respiratory medicine and potential role in asthma and COPD diagnosis. J Allergy Clin Immunol. 2021;9:2255–61. 10.1016/j.jaip.2021.02.014 33618053

[24] Feng Y Wang Y Zeng C. “Artificial intelligence and machine learning in chronic airway diseases: focus on asthma and chronic obstructive pulmonary disease.”. Int J Med Sci. 2021;18:2871–89. 34220314 10.7150/ijms.58191 PMC8241767

[25] Joumaa H Sigogne R Maravic M. “Artificial intelligence to differentiate asthma from COPD in medico-administrative databases.”. BMC Pulm Med. 2022;22:1–9. 36127649 10.1186/s12890-022-02144-2 PMC9487098

[26] Exarchos KP Beltsiou M Votti C-A. “Artificial intelligence techniques in asthma: a systematic review and critical appraisal of the existing literature.”. Eur Respir J. 2020;56:2000521. 32381498 10.1183/13993003.00521-2020

[27] Topole E Biondaro S Montagna I. “Artificial intelligence-based software facilitates spirometry quality control in asthma and COPD clinical trials.”. ERJ Open Res. 2023;9:1. 10.1183/23120541.00292-2022 PMC9907146 36776483

[28] Fernández ADR Ruiz Fernández D Gilart Iglesias V. “Analyzing the use of artificial intelligence for the management of chronic obstructive pulmonary disease (COPD).”. Int J Med Inform. 1046;158:40. 10.1016/j.ijmedinf.2021.104640 34890934

[29] Exarchos KP Aggelopoulou A Oikonomou A. “Review of artificial intelligence techniques in chronic obstructive lung disease.”. IEEE J Biomed Health Inf. 2021;26:2331–8. 10.1109/JBHI.2021.3135838 34914601

[30] Das N Topalovic M Janssens W. “Artificial intelligence in diagnosis of obstructive lung disease: current status and future potential.”. Curr Opin Pulm Med. 2018;24:117–23. 29251699 10.1097/MCP.0000000000000459

[31] Sanchez-Morillo D Fernandez-Granero MA Leon-Jimenez A. “Use of predictive algorithms in-home monitoring of chronic obstructive pulmonary disease and asthma: a systematic review.”. Chron Respir Dis. 2016;13:264–83. 27097638 10.1177/1479972316642365 PMC5720188

[32] Bećirović LS Deumic A Pokvic LG. “Aritificial intelligence challenges in COPD management: a review. 2021 IEEE 21st International Conference on Bioinformatics and Bioengineering (BIBE). IEEE, 2021.

[33] Yu G Li Z Li S. “The role of artificial intelligence in identifying asthma in pediatric inpatient setting.”. Ann Translat Med. 2020;8:1367–1367. 10.21037/atm-20-2501a PMC7723595 33313112

[34] Fernandez-Granero MA Sanchez-Morillo D Leon-Jimenez A. “An artificial intelligence approach to early predict symptom-based exacerbations of COPD.”. Biotechnol Biotechnol Equip. 2018;32:778–84.

[35] Messinger AI Luo, G Deterding RR. “The doctor will see you now: How machine learning and artificial intelligence can extend our understanding and treatment of asthma.”. J Allergy Clin Immunol. 2020;145:476–8. 31883444 10.1016/j.jaci.2019.12.898 PMC7035143

[36] Badnjevic A Gurbeta L Custovic E. “An expert diagnostic system to automatically identify asthma and chronic obstructive pulmonary disease in clinical settings.”. Sci Rep. 2018;8:11645. 30076356 10.1038/s41598-018-30116-2 PMC6076307

[37] Spathis D Vlamos P. “Diagnosing asthma and chronic obstructive pulmonary disease with machine learning.”. Health Informatics J. 2019;25:811–27. 28820010 10.1177/1460458217723169

[38] Mekov E Miravitlles M Petkov R. “Artificial intelligence and machine learning in respiratory medicine.”. Expert Rev Respirat Med. 2020;14:559–64. 32166988 10.1080/17476348.2020.1743181

[39] Antão J de Mast J Marques A. “Demystification of artificial intelligence for respiratory clinicians managing patients with obstructive lung diseases.”. Expert Rev Respirat Med. 2024;17:1–13. 10.1080/17476348.2024.2302940 38270524

[40] Gelman A Sokolovsky V Furman E. Artificial intelligence in the respiratory sounds analysis and computer diagnostics of bronchial asthma. medRxiv. 2021;17:2021–11.

[41] Hashimoto DA Witkowski E Gao L. Artificial Intelligence in anesthesiology: current techniques, clinical applications, and limitations. Anesthesiology. 2020;132:379–94. 31939856 10.1097/ALN.0000000000002960 PMC7643051

[42] Hunter B Hindocha S Lee RW. The role of Artificial Intelligence in Early cancer diagnosis. Cancers (Basel). 2022;14:1524. 35326674 10.3390/cancers14061524 PMC8946688

[43] Ji Y Ji Y Liu Y. Research progress on diagnosing retinal vascular diseases based on artificial intelligence and fundus images. Front Cell Dev Biol. 2023;11:1168327. 37056999 10.3389/fcell.2023.1168327 PMC10086262

